# LC3B globular structures correlate with survival in esophageal adenocarcinoma

**DOI:** 10.1186/s12885-015-1574-5

**Published:** 2015-08-12

**Authors:** Shereen El-Mashed, Tracey R. O’Donovan, Elaine W. Kay, Ayat R. Abdallah, Mary-Clare Cathcart, Jacintha O’Sullivan, Anthony O’Grady, John Reynolds, Seamus O’Reilly, Gerald C. O’Sullivan, Sharon L. McKenna

**Affiliations:** 1 Leslie C. Quick Laboratory, Cork Cancer Research Centre, BioSciences Institute, University College, Cork, Ireland; 2 Department of Pathology, Royal College of Surgeons Ireland (RCSI), Beaumont Hospital, Dublin, Ireland; 3 National Liver Institute, Menoufiya University, Shebin El Kom, Egypt; 4 Department of Surgery & Trinity Centre for Health Sciences, St James Hospital, Dublin, Ireland; 5 Department of Oncology, Cork University Hospital, Cork, Ireland

## Abstract

**Background:**

Esophageal adenocarcinoma has the fastest growing incidence of any solid tumor in the Western world. Prognosis remains poor with overall five-year survival rates under 25 %. Only a limited number of patients benefit from chemotherapy and there are no biomarkers that can predict outcome. Previous studies have indicated that induction of autophagy can influence various aspects of tumor cell biology, including chemosensitivity. The objective of this study was to assess whether expression of the autophagy marker (LC3B) correlated with patient outcome.

**Methods:**

Esophageal adenocarcinoma tumor tissue from two independent sites, was examined retrospectively. Tumors from 104 neoadjuvant naïve patients and 48 patients post neoadjuvant therapy were assembled into tissue microarrays prior to immunohistochemical analysis. Kaplan-Meier survival curves and log-rank tests were used to assess impact of LC3B expression on survival. Cox regression was used to examine association with clinical risk factors.

**Results:**

A distinct globular pattern of LC3B expression was found to be predictive of outcome in both patient groups, irrespective of treatment (*p* < 0.001). Multivariate analysis found that this was a strong independent predictor of poor prognosis (*p* < 0.001).

**Conclusions:**

This distinctive staining pattern of LC3B represents a novel prognostic marker for resectable esophageal adenocarcinoma.

**Electronic supplementary material:**

The online version of this article (doi:10.1186/s12885-015-1574-5) contains supplementary material, which is available to authorized users.

## Background

The last two decades have seen a significant increase in the incidence of cancer of the oesophagus, with it becoming the seventh leading cause of cancer death in the Western world [1]. There are two main histological types of esophageal cancer; squamous cell carcinoma and adenocarcinoma. The increasing incidence is predominantly in esophageal adenocarcinoma. The principal treatment regimen for localized esophageal adenocarcinoma in Europe is pre-operative chemotherapy which is based on cisplatin/5-fluorouracil (5-FU) or radio-chemotherapy, followed by surgical resection [2]. Despite improvements in diagnosis and treatment approaches, overall five-year survival rates remain under 25 % [3]. Current approaches cause considerable toxicity in the vast majority of patients and there are no pre-therapy markers that could help to tailor treatment to those who would benefit the most [4]. Pathological classifications of tumor grade, differentiation, vascular invasion and lymph node status are sub-optimal in predicting response to neoadjuvant therapy [5]. Even following complete pathological response, there is a significant risk of disease recurrence and cancer-specific death following resection [6].

Basal and dynamic levels of autophagy are responsible for the degradation of long lived or aggregated proteins and damaged or superfluous organelles. Autophagy is initiated with the formation of a double-membraned phagophore that encapsulates cellular material and extends to become a vesicle referred to as an autophagosome [7]. This then fuses with the lysosome, enabling degradation of its contents [8]. Elongation of the phagophore membrane incorporates LC3II (microtubule-associated protein Light Chain 3) which is considered the most specific marker for autophagosome formation (and autophagy) [9].

Autophagy has been implicated in the pathogenesis of cancer [10], but its exact role is still a matter of debate as autophagy has been associated with both better and poorer outcome [11, 12]. Autophagy may act as a survival mechanism and energy reservoir for tumors subjected to stressful conditions such as; hypoxia, nutrient deprivation, metabolic stress and chemotherapy [13–16]. Inhibition of autophagy has been reported to chemosensitize several otherwise resistant cancer cells including chronic myeloid leukemia [17], ovarian cancer [18], breast cancer [19], malignant glioma [20] and esophageal cancer [21]. Conversely, promotion of autophagy has been reported to be important for anti-tumor immune responses following chemotherapy [22, 23].

Several studies have evaluated the potential prognostic value of autophagy markers, including Beclin 1 and LC3. Most studies of LC3 have examined overall expression levels and there is divergent opinion on its clinical significance (see later/discussion). In addition, other studies have reported distinct distribution patterns of the autophagy marker LC3 in several cancers. In particular, staining of LC3A stone-like structures (SLS) has been associated with tumor progression and a poor prognosis in epithelial tumors [24]. In gastric cancers, these structures were associated with an increased rate of recurrence after resection [25]. The significance of LC3 staining patterns in esophageal adenocarcinoma is still unknown. In this study, we evaluated the expression of the autophagy marker LC3B in two different groups of esophageal adenocarcinoma patients; Group 1 (neoadjuvant-naïve) and Group 2 (received pre-operative neoadjuvant therapy). We examined the relationship between LC3B expression and overall survival of patients in both groups.

## Methods

### Patients

Patients who were diagnosed with esophageal adenocarcinoma were identified retrospectively from the pathology department records at the Mercy University Hospital, Cork (*n* = 50) and Saint James’s Hospital, Dublin, Ireland (*n* = 102). Ethical approval for this study was obtained from the Clinical Research Ethics Committee of the Cork Teaching Hospitals and for the St. James site, the Ethics Committee at the Adelaide and Meath Hospital, Tallaght, Dublin. All of the patients were diagnosed and treated at the Mercy University Hospital, Cork or St James’s Hospital, Dublin, between 2000 and 2006. One hundred and four patients did not receive neoadjuvant therapy (Group 1) while 48 patients received neoadjuvant therapy (Group 2). All neoadjuvant patients in Cork and Dublin had pre-operative chemo-radiotherapy, followed by surgical resection. Neoadjuvant treatment is indicated for patients who are candidates for curative surgical resection. Chemotherapy is based on cisplatin/5-fluorouracil (5-FU). Clinical and histopathological data from both sites is shown in Additional file 1: Table S1. Archival formalin fixed, paraffin embedded tumor blocks and corresponding normal esophageal mucosa, and lymph node metastasis (if available), were also collected. All surgical esophageal adenocarcinoma tumors were assembled into tissue microarrays (TMAs) prior to immunohistochemical (IHC) analysis.

**Table 1:** Relationship between distinct LC3B staining patterns in esophageal adenocarcinoma patient Group 1 (neoadjuvant-naïve). Statistical analysis was carried out using chi-squared test (* *p* < 0. 05)

	LC3B globular structures			LC3B cytoplasmic		
	Negative	Positive	*p*–value	Negative	Positive	*p*-value
LC3B crescent or ring-like structures						
Negative	50(90.9 %)	18(36.7 %)	<0.001*	36(61.0 %)	32(71.1 %)	0.3
Positive	5(9.1 %)	31(63.3 %)		23(39.0 %)	13(28.9 %)	
LC3B cytoplasmic						
Negative	20(36.4 %)	39(79.6 %)	<0.001*			
Positive	35(63.6 %)	10(20.4 %)				

### Cell culture

Established human esophageal cancer cell lines OE19, OE21 and OE33, were obtained from the ECACC (European Collection of Cell Cultures, 96071721, 96062201 and 96070808). KYSE450 cells were from DSMZ (Deutsche Sammlung von Mikroorganismen und Zellkulturen GmbH). OE21 and KYSE450 are of squamous origin and OE19 and OE33 are adenocarcinoma. OE19, OE21 and OE33 cell lines were maintained in RPMI 1640 medium, KYSE450 cells were maintained in 50:50 RPMI 1640:F-12 HAMS medium, all supplemented with 1 % penicillin/streptomycin, 10 % (*v/v*) fetal calf serum (Gibco, 21875–034, 15070–063, 10270) at 37 °C, 5 % CO_2_.

### Embedding cell lines into paraffin

Cells (~2.0 × 10^7^) were collected, washed with PBS and re-suspended in 1 ml of warm 1 % agarose/10 % neutral buffered formalin. The pellets were then processed by standard tissue processing technique and embedded into a paraffin block.

### Analysis of recombinant proteins by western blotting

Human recombinant microtubule-associated proteins 1A/1B light chain 3A and 3B were purchased from Enzo Life Sciences (BML-UW1145 and BML-UW1155, respectively) and run on NuPAGE 4–12 % Bis-Tris gels (Invitrogen NP0322) and probed with either the Abgent anti-LC3 antibody (Abgent AP1802a) or with the MBL anti-LC3A & B antibody (MBL PD014) to confirm integrity of protein. Proteins were visualized using relevant IR-Dye conjugated secondary antibodies (Li-Cor 926–32211) on the Odyssey IR imaging system (Li-Cor, UK). Quantified data is presented as integrated intensity.

### Immunofluorescence staining

Cytospins were fixed in 4 % paraformaldehyde (PFA) for 20 min and washed with PBS. Permeabilization was carried out with 0.005 % saponin/PBS for active caspase-3 and 0.2 % Triton X for LC3B. All sections were blocked with 5 % BSA/PBS for 1 h at room temperature. Primary antibodies [LC3B (Abgent, AP1802a) and active caspase-3 (Cell Signaling 9664)] were incubated overnight at 4 °C. After incubation with alexa fluor 488 (Invitrogen, Ireland) slides were mounted with Prolong Gold antifade reagent with DAPI (Molecular Probes, P36935). All slides were viewed using a DP70 Olympus digital microscope camera at 40×. Images were captured with Olympus DP-Soft823 version 3.2 acquisition software.

### Immunohistochemistry staining

Slides were de-waxed and antigen retrieval was performed in 0.1 M citrate buffer (pH 6.0) with microwave heating for 20 min. Endogenous peroxidase was blocked with peroxidase block (EnVision + System-HRP (DAP), Dako) for 5 min, at room temperature, and 5 % BSA in PBS was added to the slides for 1 h to reduce nonspecific binding. Slides were incubated overnight at 4 °C with Atg8b (LC3B) antibody (Abgent AP1802a) diluted 1:100 in 5 % BSA/PBS. After washing in PBS, sections were incubated with polymer-HRP labelled secondary antibody (EnVision + System-HRP (DAP), Dako) at 37 °C for 45 min. A drop of DAP-chromagen was added and sections were counterstained with hematoxylin. In negative control slides, PBS replaced the primary antibody and positive staining was absent.

### Quantification of immunohistochemistry staining

Three distinct patterns of LC3B immunohistochemical staining were identified and enumerated in each tissue core (subsequently referred to as sections); (i) cytoplasmic, (ii) crescent or ring-like structures and (iii) large globule like structures.

These three patterns of LC3B staining were assigned scores as follows: The proportion of neoplastic cells with a cytoplasmic pattern of reactivity ranged from 5–90 % per section at 100× magnification (median, 40 %). The 60th percentile was used to classify tumours into negative (< 60 % percentile) and positive (≥ 60 % percentile). Tumor sections were considered positive if 50 % or more of the viable tumor cells showed strong cytoplasmic staining.

The number of crescent or ring-like structure and globular structures were enumerated in each section at a magnification of 400× and expressed as the mean of all counts. The number of crescent or ring-like structures and globular structures ranged from one to six (80th percentile was four) per section. The tumors were subsequently classified according to 80th percentile into negative (< 80th percentile) versus positive (≥ 80th percentile). Counting four structures or more, in five independent fields of view, was used to classify tumors as positive, while less than four were classified as negative. In addition, receiver operating characteristic (ROC) curve analysis was performed to confirm this cut off value for positivity. All slides were viewed using a DP70 Olympus digital microscope camera at 100×, 400× and 1000× (Mason Technologies, UK). Images were captured with Olympus DP-Soft823 version 3.2 acquisition software. IHC scores were assessed independently by two pathologists (S.E-M. & E.K., *authors*) who were blinded to patient clinical data. Scoring was consistent in 85 % of cases. Inconsistent scores were reassessed by both S.E-M. and E.K. to assign final score.

### Clinicopathologic data and outcome

The following clinical and histopathological data were collected from medical charts and pathology reports: age, gender, neoadjuvant therapy, follow up, tumor stage and differentiation, vascular and neural invasion. The clinical and histopathological data in both esophageal adenocarcinoma patient groups are shown in Additional file 1: Table S1.

### Statistical analysis

Statistical analysis was carried out using SPSS software (SPSS Inc., version 19; USA). Receiver operating characteristic curve analysis was plotted using SPSS to determine the cut off points for scoring. Chi-square test was used to measure the association between qualitative variables. Fisher exact test was used for 2 × 2 qualitative variables where more than 25 % of the cells have an expected count of less than 5. Kaplan-Meier survival curves were used to assess impact of variables on overall survival. Where appropriate, Cox regression was used to give an adjusted hazard ratio and 95 % confidence interval of the effect of the different risk factors for survival. The *p*-value was considered statistically significant when it was less than 0.05. Patients form Cork and Dublin were analyzed separately and then together – as both patient cohorts achieved significance with the same pattern.

## Results

### Validation of the LC3B autophagy marker

**Fig. 1:**
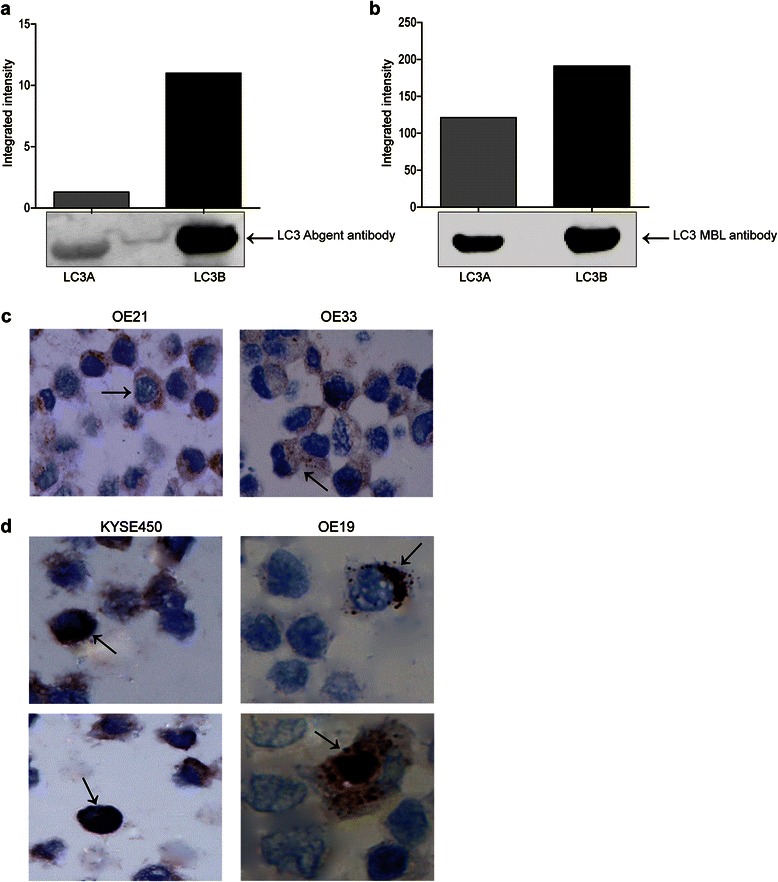
Western blot analysis of human recombinant microtubule-associated proteins 1A/1B light chain 3A and 3B probed with either **a** the Abgent anti-LC3 antibody (*AP1802a*), or **b** MBL anti-LC3A/B antibody. LC3 bands were quantified using the Odyssey Infrared Imaging System (*Li-Cor*), and data presented as integrated intensities (*n* = 3). **c** Analysis of LC3B distribution in untreated OE21 and OE33 esophageal cancer cell lines. Arrows indicate diffuse cytoplasmic LC3B expression (*magnification 400×*) (*n* = 6). **d** Analysis of LC3B distribution in untreated KYSE450 and OE19 esophageal cell lines. Arrows indicate the presence of LC3B crescent or ring-like (*upper panels*) and globular (*lower panels*) structures in KYSE450 and OE19 cells (*magnification 1000×*) (*n* = 6)

Prior to investigating expression of the autophagy marker in cell lines or patient samples, we evaluated the specificity of the Abgent LC3B antibody by western blotting with recombinant proteins. Western blot analysis of both LC3B and LC3A recombinant proteins confirmed that the Abgent LC3B antibody had a very weak affinity for LC3A and predominantly reacts with LC3B (Fig. 1a). In contrast, an MBL LC3 dual A/B reactive antibody detected both isoforms of LC3 (Fig. 1b). This analysis confirmed high specificity of the Abgent antibody for the LC3B isoform.

Previous work from this laboratory had identified drug sensitive (OE21 and OE33) and resistant esophageal cell lines (KYSE450 and OE19) that respond to therapeutic agents by inducing apoptosis and autophagy respectively [21]. Prior to investigating patient samples, we first validated immunohistochemical (IHC) detection of LC3B in esophageal cancer cell lines, pre- and post-treatment with the chemotherapeutic drug 5-fluorouracil (5-FU). Standard IHC staining shows the expression of LC3B in paraffin embedded OE21 and KYSE450 cell lines (Additional file 2: Figure S1A and B). OE21 (apoptotic competent) cells show mild staining of LC3B before and after treatment (Additional file 2: Figure S1A). In contrast, KYSE450 cells show mild LC3B staining in pre-treatment sections, whereas strong staining is evident following 5-FU treatment (Additional file 2: Figure S1B). This corresponds to immunofluorescence (IF) staining for LC3B in non-embedded cell lines (Additional file 2: Figure S1C and D, respectively).

### Distribution of LC3B staining in untreated cells

Expression of LC3B was also evaluated by IHC in untreated drug sensitive (OE21 and OE33) and resistant esophageal cell lines (KYSE450 and OE19) (Fig. 1c and d, respectively). The LC3B staining detected in both cell lines had a specific distribution, which was obvious at higher magnification (400× and 1000×). Three distinct patterns of LC3B expression were noted: (1) diffuse cytoplasmic pattern, (2) crescent or ring-like structures and (3) large globular structures. The first pattern; diffuse cytoplasmic expression, which consists of fine granular structures distributed heterogeneously, was the predominant staining pattern in untreated OE21 and OE33 (drug sensitive) cells (Fig. 1c *arrows*). The other two patterns were found in untreated KYSE450 and OE19 (drug resistant) cells. In ~ 1/40 cells, a crescent shaped LC3B stained structure at the nucleus or a perinuclear ring structure, was evident (Fig. 1d *upper panels, arrows*). This appears to be formed from a cluster of other smaller structures at the periphery of the nucleus. In ~ 1/80 cells, a large globular structure was present and this often occupied the majority of the cell, marginalising the nucleus (Fig. 1d *lower panels, arrows*).

### Immunohistochemical analysis of LC3B expression in esophageal adenocarcinoma patient tissue

**Fig. 2:**
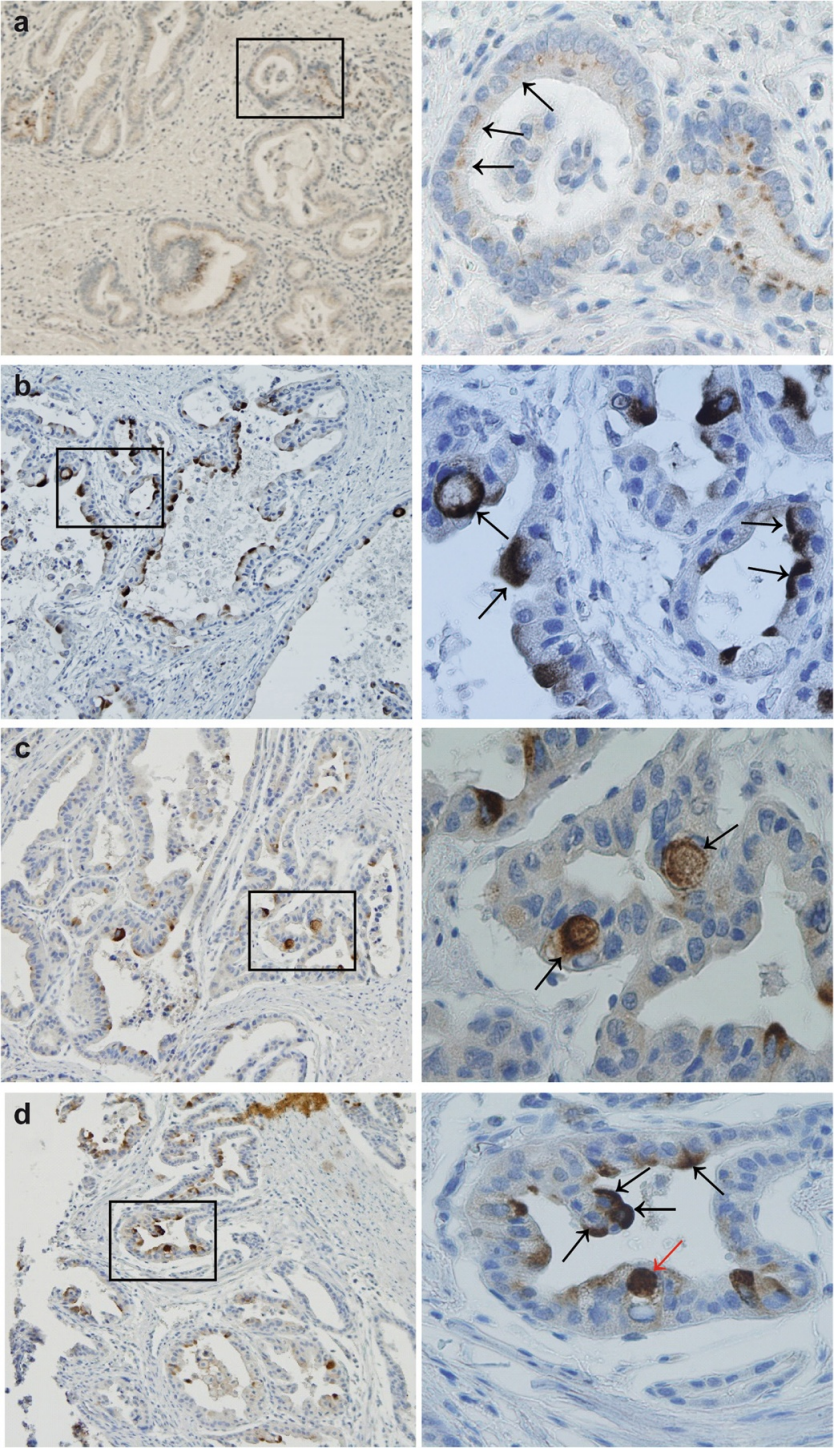
LC3B expression patterns in esophageal adenocarcinoma tumor samples. All images to the left are at a magnification of 100×; the area within the rectangle is shown to the right at the higher magnification of 400×. **a** Representative images of cytoplasmic expression of LC3B in esophageal adenocarcinoma tumor cells (black arrows). **b** LC3B crescent or ring-like structures in tumor cells (*black arrows*). **c** LC3B globular structures (*black arrows*). **d** LC3B crescent or ring-like structures (*black arrows)* and a globular structure which appears to be within a vacuole (*red arrow*)

The same three patterns of LC3B were also observed in patient tumor samples (Fig. 2a-d): (i) cytoplasmic pattern, (ii) crescent or ring-like structures and (iii) large globule like structures. All patient samples (Group 1 and 2) were scored and analyzed statistically, according to the following LC3B staining distributions.

(i) Cytoplasmic
  A cytoplasmic, finely granular or dot-like LC3B staining pattern was observed in tumor sections from both groups. In contrast to the diffuse distribution observed in the cell lines, the staining in patient samples was more polarized towards the apical side of the cell (representative images shown in Fig. 2a *black arrows*). Tumor sections were considered positive if 50 % or more of the viable tumor cells showed strong cytoplasmic staining. In Group 1 (neoadjuvant-naïve), 43.3 % of tumor sections showed positive LC3B cytoplasmic staining. This distribution of staining was similar in Group 2 (neoadjuvant therapy) where 52.1 % of tumor sections were also positive.
(ii) Crescent or ring-like structure
  The crescent or ring-like structure, composed of multiple LC3B positive dots, was identified at the periphery of the tumor cell nucleus in esophageal adenocarcinoma tumor sections (representative images shown in Fig. 2b and d *black arrows*). The number of crescent or ring-like structures ranged from 1 to 6 per section. The crescent or ring-like structures in each tumor section were enumerated and tumor samples were then classified into groups accordingly. Four or more than four crescent or ring-like structures per section was classified as positive staining. In Group 1 (neoadjuvant-naïve), 34.6 % of tumor sections were scored as positive, while the remaining sections had less than four structures in the examined section. In Group 2 (neoadjuvant therapy), up to 72.9 % of tumor sections were positive for crescent or ring-like structures.
(iii)  Globular structure
  A globular, densely stained structure was the third LC3B staining pattern observed. These globular structures varied in size, but usually they were large, with some occupying most of the cytoplasm, flattening the nuclei toward the periphery (representative images shown in Fig. 2c *black arrows*) or occasionally appearing to be within a vacuole (Fig. 2d *red arrow*). The esophageal adenocarcinoma patient tumor samples were then grouped into positive and negative as above, with four or more globular structures, in five independent fields of view being classified as positive staining. In patient Group 1 (neoadjuvant-naïve), 47.1 % of tumor samples displayed positive staining of globular LC3B structures, whereas in Group 2 (neoadjuvant therapy), positive staining was observed in 77.1 % of tumor samples.

### Relationship between LC3B staining patterns and survival

Kaplan-Meier survival curves were plotted to assess whether there is an association between LC3B staining patterns and patient outcome. Patients from the two different sites, Cork (*n* = 50) and Dublin (*n* = 102), were initially analyzed separately and then together for all parameters, as both patient cohorts achieved similar significance with the same assessment.

**Fig. 3:**
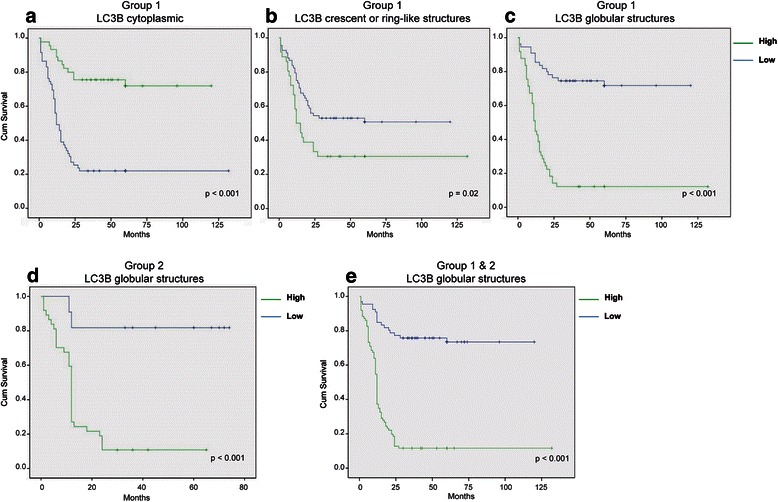
Relationship between LC3B staining patterns and survival in patient Group 1 and Group 2. Kaplan-Meier survival curves for **a** cytoplasmic, **b** crescent or ring-like structures and **c** globular LC3B staining patterns in Group 1 patients (neoadjuvant-naïve). **d** Kaplan-Meier survival curves for LC3B staining patterns in Group 2 patients (*neoadjuvant therapy*). **e** Collective survival analysis of globular structures in patient Groups 1 and 2

In Group 1 patients (neoadjuvant-naïve), positive cytoplasmic reactivity to LC3B was predictive of favourable outcome, with > five-year survival, when compared with negative cytoplasmic staining [(*p* < 0.001) (Fig. 3a)]. Group 1 patients with negative LC3B crescent or ring-like structures had a better prognosis when compared to patients with positive staining [(*p* = 0.02) (Fig. 3b)]. These staining patterns were not predictive of prognosis in Group 2 patients (data not shown). A higher incidence of LC3B positive globular structures, was highly predictive of poorer outcome in both Group 1 [(*p* < 0.001) (Fig. 3c)] and Group 2 [(*p* < 0.001) (Fig. 3d)] patients. As this marker predicts five year survival, regardless of whether the patient received neoadjuvant therapy or not, Group 1 and Group 2 patients can be pooled and collectively assessed (Fig. 3e). These structures are highly indicative of outcome, irrespective of treatment (*p* < 0.001).

### Association between LC3B immunostaining patterns

The origin of the LC3B staining patterns is unknown and it is possible that they are related or represent a functional status of the tumor cell. We examined whether there was any association between the cytoplasmic, crescent or ring-like and globular LC3B staining patterns. In Group 2 patients (neoadjuvant therapy), there was no significant association between the staining patterns. In contrast, in Group 1 patients (neoadjuvant-naïve), the LC3B globular structures had a direct relationship with crescent or ring-like structures (Table 1). In patients who are positive for LC3B globular structures, 63.3 % of these are also positive for LC3B crescent or ring-like structures (*p* < 0.001) (see also representative image Fig. 2d). The formation of these structures may therefore be inter-related. Conversely, there was an indirect relationship between LC3B globular structures and the cytoplasmic pattern of LC3B. Of those patients who were negative for LC3B globular structures, 63.6 % also had positive LC3B cytoplasmic staining (*p* < 0.001). It is possible that the cytoplasmic LC3B staining is present in a more normal/less stressed state that does not tend to aggregate LC3B into larger more defined structures. There was no significant association between the cytoplasmic LC3B pattern and crescent or ring-like structures. It is not known why these associations are only in Group 1 (neoadjuvant-naïve).

### Multivariate analysis

We then examined the independent prognostic value of the LC3B globular structure in both groups of esophageal adenocarcinoma patients by multivariate cox regression analysis. Multivariate analysis of parameters commonly associated with poor prognosis, including tumor differentiation, tumor staging, lymph node metastasis, vascular invasion, neural invasion and LC3B globular structures is shown in Table 2. In group 1 (neoadjuvant-naïve); the presence of LC3B positive globular structures (HR = 6.086; 95 % confidence interval (CI) = 3.179–11.653; *p* < 0.001) and vascular invasion (HR = 2.304; 95 % confidence interval (CI) = 1.211–4.384; *p* = 0.011) were independent predictors of poor prognosis in all the variables examined. In group 2 (neoadjuvant therapy); LC3B positive globular structures was the only independent predictor of poor prognosis in all the variables examined (HR = 9.136; 95 % confidence interval (CI) = 2.115–39.462; *p* = 0.003).

**Table 2:** Multivariate analysis of LC3B globular structures and other clinical pathological parameters that may have a potential role in prognosis. The presence of LC3B globular structures has a strong independent prognostic value (*p* < 0.001)

	Group 1 patients (neoadjuvant-naïve)		Group 2 (neoadjuvant therapy)	
	Hazard ratio (95 % CI)	*p*-value	Hazard ratio (95 % CI)	*p*-value
LC3B globular structures				
Negative v’s Positive	6.086 (3.179–11.653)	<0.001*	9.136 (2.115–39.462)	0.003*
Differentiation				
Mild*	1(−)	0	1(−)	0
Moderate	0.667 (0.213–2.086)	0.487	0.000	0.976
Poor	0.898 (0.516–1.564)	0.704	0.677 (0.318–1.441)	0.311
Tumor staging				
Stage I*	1(−)	0	1(−)	0
Stage II	0.627 (0.193–2.029)	0.435	2.877 (0.323–25.59)	0.343
Stage III-IV	0.858 (0.204–3.608)	0.835	5.043 (0.457–55.67)	0.187
Lymphatic mets				
Negative v’s Positive	1.632 (0.504–5.288)	0.414	0.634 (0.196–2.055)	0.448
Vascular invasion				
Negative v’s Positive	2.304 (1.211–4.384)	0.011*	0.559 (0.220–1.416)	0.220
Neural invasion				
Negative v’s Positive	1.050 (0.548–2.009)	0.883	2.327 (0.876–6.181)	0.090

### Correlation between LC3B staining and histopathological parameters

We also investigated whether LC3B staining was associated with histopathological parameters and tumor aggressiveness. We examined the relationship between LC3B distribution patterns and tumor differentiation, tumor staging and tumor metastasis in lymph nodes, blood vessels or nerve fibres. An association with histopathological parameters was found in Group 1 (neoadjuvant-naïve) patients, when LC3B staining patterns were analyzed independently (Table 3). Negative LC3B cytoplasmic reactivity is associated with esophageal adenocarcinoma of moderate to poor differentiation (*p* = 0.015). In addition, negative LC3B cytoplasmic reactivity is associated with lymph node tumor metastasis (*p* = 0.001), tumors with a late stage (III-IV) (*p* = 0.001) and neural invasion (*p* = 0.004). On the other hand, positivity for LC3B globular structures is associated with lymph node tumor metastasis (*p* = 0.013) and tumors with a late stage (III-IV) (*p* = 0.003). In Group 2 (neoadjuvant therapy), no association was detected between LC3B and the aforementioned histopathological parameters (data not shown). It is possible that the higher overall levels of LC3B staining, as a consequence of neoadjuvant therapy in Group 2 may obscure normal basal levels that may have shown a relationship. This may also be due to a difference in tumor biology following treatment.

**Table 3:** Relationship between LC3B staining patterns and histopathological parameters in Group 1 (neoadjuvant-naïve) esophageal adenocarcinoma patients. Statistical analysis was carried out using chi-squared test (* *p* < 0. 05)

	LC3B Cytoplasmic			LC3B Globular structures		
	Negative	Positive	*p*–value	Negative	Positive	*p*–value
Differentiation						
Well	3(5.1 %)	8(17.8 %)	0.015*	8(14.5 %)	3(6.1 %)	0.292
Moderate	29(49.2 %)	27(60.0 %)		30(54.5 %)	26(53.1 %)	
Poor	27(45.8 %)	10(22.2 %)		17(30.9 %)	20(40.8 %)	
Tumor staging						
Stage I	8(13.6 %)	9(20.0 %)	0.001*	12(21.8 %)	5(10.2 %)	0.003*
Stage II	9(15.3 %)	20(44.4 %)		21(38.2 %)	8(16.3 %)	
Stage III-IV	42(71.2 %)	16(35.6 %)		22(40.0 %)	36(73.5 %)	
Lymphatic mets						
Negative	11(18.6 %)	23(51.1 %)	0.001*	24(43.6 %)	10(20.4 %)	0.013*
Positive	48(81.4 %)	22(48.9 %)		31(56.4 %)	39(79.6 %)	
Vascular invasion						
Negative	34(57.6 %)	29(64.4 %)	0.546	36(65.5 %)	27(55.1 %)	0.319
Positive	25(42.4 %)	16(35.6 %)		19(34.5 %)	22(44.9 %)	
Neural invasion						
Negative	36(61.0 %)	39(86.7 %)	0.004*	41(74.5 %)	34(69.4 %)	0.558
Positive	23(39.0 %)	6(13.3 %)		14(25.5 %)	15(30.6 %)	

### LC3B expression in non-neoplastic esophageal squamous epithelium

In Group 1 patients (neoadjuvant-naïve), no LC3B staining was detected in non-neoplastic esophageal squamous epithelium. While Group 2 (neoadjuvant therapy) showed low LC3B cytoplasmic reactivity. Crescent or ring-like and globular LC3B structures were not detected in normal esophageal squamous epithelium in either group (data not shown).

## Discussion

In this study, we have shown that specific staining patterns of the autophagy marker (LC3B) are associated with patient outcome in esophageal adenocarcinoma.

The distribution patterns of LC3B were also relevant in esophageal cell lines. Diffuse cytoplasmic LC3B staining was detected in untreated, drug sensitive cell lines (OE21 and OE33) while the other two patterns; the crescent or ring-like, and the globular patterns were present only in drug resistant cells (KYSE450 and OE19). The LC3B expression patterns in patient samples corresponded with the cell lines. In patients who did not receive therapy prior to surgical resection, a positive cytoplasmic LC3B pattern was statistically correlated with a more favourable outcome, whereas the crescent or ring-like and the globular patterns were associated with poor outcome. Importantly, the globular pattern is associated with unfavourable prognosis, regardless of whether the patient had neoadjuvant therapy or not. Multivariate analysis found this marker to be the strongest independent predictive variable in esophageal cancer. This type of marker could be extremely useful, as outcome could be determined in advance of a treatment that is only effective in a sub-group of patients.

A relationship between the two patterns of LC3B staining, crescent or ring-like and the globular staining pattern was also identified suggesting that they may originate from the same process or be associated with the same phenotype. Positive cytoplasmic LC3B staining was also associated with a well-differentiated tumor, while negative cytoplasmic expression was associated with late tumor stage and lymph node metastasis. In addition, negative crescent/globular structures were associated with absence of vascular invasion. These results raise the possibility that the crescent/globule patterns represent a new phenotype which supports tumor progression. It is notable that these globule structures were only seen in a sub-population of the cells, in both the cell lines and patient samples, the reason for this is not known.

### Studies of overall expression levels of LC3

Other studies have evaluated overall expression of LC3 in cancer, with variable conclusions regarding significance. A study of pancreatic tumors reported that high diffuse cytoplasmic LC3 reactivity at the periphery of the tumor correlated with shorter disease free survival. A relationship was identified between LC3 expression, tumor size and tumor necrosis. The antibody used was anti-LC3A on tissue sections from 71 cases [26]. LC3 expression was also examined in gastrointestinal cancer including esophageal squamous carcinoma and no significant correlation between LC3 expression and various clinicopathological parameters or overall survival was identified [27]. In contrast, another study of esophageal squamous cell carcinoma reported that a high overall level of LC3, detected with a Novus rabbit antibody (NB100-2220) is associated with shorter survival [28]. In glioblastoma, high positive cytoplasmic staining of LC3B was associated with an improved outcome in patients [29]. A study of colorectal cancer, using an Abcam LC3B polyclonal antibody, found negative LC3B expression and absence of autophagy related proteins (Beclin 1 ATG5 LC3B) are associated with poor survival [30]. An analysis of LC3A (Abcam EP1528Y) and LC3B (Abcam polyclonal) expression in breast cancer found an association with triple negative and high grade tumors, but no overall association with prognosis [31]. A meta-analysis of five LC3B expression studies (scoring high & low) in breast cancer reported that a high expression of LC3B predicted a greater risk of mortality [32]. While it is possible that these different conclusions reflect different tissue types, there is no uniformity in the procedures and reagents (LC3A or LC3B antibodies from various sources). The isoform selectivity of many of these antibodies is currently unclear and the role of the five LC3 isoforms (LC3Av1, LC3Av2, LC3B, LC3B2 and LC3C) and related family members (GATE16 and GABARAP) in autophagy has yet to be determined. Importantly, there are divergent scoring methods and an absence of clear criteria of positivity for the LC3 markers.

### Studies reporting ‘stone like structures’ (SLS)

A number of studies from one group have reported LC3 data analogous to our data in other epithelial cancers. They have reported distinct LC3A expression patterns in breast [33], endometrial [34], pulmonary carcinoma [35], cutaneous SCC [36], urothelial cell carcinoma (UCC) [37] and colorectal cancer [38], using an anti-LC3A antibody from Abgent (AP1805a). This antibody detects recombinant LC3A and not LC3B [39]. They detected LC3A positive ‘stone like’ structures (SLS) that were strongly associated with tumor aggressiveness and poor prognosis. These resemble the LC3B globular structures that we have identified as a strong prognostic marker in esophageal adenocarcinoma. They found that prognosis was largely unaffected by the diffuse cytoplasmic pattern, except in UCC where extensive cytoplasmic staining was associated with muscle invasion [37]. In colorectal and breast cancer a juxta-nuclear accumulation of LC3A protein was associated with lack of metastasis and predicted good prognosis.

In hepatocellular carcinoma, expression of LC3 at advanced tumor stages (but not early stages) was correlated with longer survival. Diffuse cytoplasmic, juxta nuclear and ‘stone like’ structures were also examined and were not associated with prognosis in this study. This study employed an LC3 antibody from Novus Biologicals [40] and they found few patients with ‘stone like’ LC3 expression. Conversely, another group employing a rabbit polyclonal anti-LC3A antibody (Abcam) reported a ‘stone like’ pattern of LC3A expression to be an independent, highly prognostic factor in hepatocellular carcinoma [41]. Another group also identified LC3A SLS with the Abgent LC3A antibody (AP1805a) in gastric cancer. A high number of SLS was associated with increased risk of recurrence after resection of stages -III and lower overall survival rate for stage IV [25].

Thus, the use of antibodies directed to different isoforms of LC3 (LC3A - SLS studies and LC3B – our group) enabled similar conclusions to be drawn by most groups regarding the prognostic significance of LC3 globules/SLS in patient tissue. We currently do not know if the Abgent LC3A antibody would have produced similar data to LC3B in our samples. A recent study has reported that LC3Av1 and LC3B are the most ubiquitously expressed isoforms in normal human tissue and LC3Av1 is frequently silenced in cancer [42]. Indeed, in OSCC the LC3Av1 gene is silenced in 66.7 % (20/30) of cell lines analyzed. Therefore, if this was reflected in esophageal adenocarcinoma and we had used an antibody specific for LC3A rather than LC3B we may have missed a relationship. It is also interesting that this group reported silencing of LC3Av1 in 21 % of lung, 80 % of endometrial and 25 % of colorectal cancer cell lines. However, LC3A SLS were detected in these cancers by the Koukourakis team, suggesting that this silencing may not happen *in vivo,* or that the LC3Av2 may compensate for loss of LC3Av1. It is also possible that both LC3A and LC3B are incorporated into these globular/SLS structures and that the LC3B antibody in our study and the LC3A antibodies in the SLS studies are detecting the same structures.

The origin of these morphologically distinct LC3 stained structures is unknown. The more diffuse staining in the cytoplasm may reflect soluble LC3B and basal physiological autophagic activity. If these cancer cells have a more ‘normal’ physiology – this may explain the better prognosis. In contrast, the LC3A/B globular pattern is an exclusive finding of aggressive malignant epithelial cancers and may reflect an exaggerated or aberrant form of autophagic activity. Further research is needed to investigate the nature of this structure, which is clearly linked to an aggressive tumor phenotype.

In conclusion, this is the first analysis of autophagy markers in esophageal adenocarcinoma. In particular, the LC3B globular structures identified in esophageal adenocarcinoma patients are strongly associated with patient outcome irrespective of treatment. The results of this study are consistent with current published data on other cancers and suggest that LC3B may be a unique marker in predicting prognosis in esophageal cancer. Further multi-centre retrospective and prospective studies, would be encouraged to further validate the prognostic significance of this marker.

## Additional files

Additional file 1: Table S1. Clinical and histopathological data from both Group 1 (neoadjuvant-naïve) and Group 2 (neoadjuvant therapy) esophageal adenocarcinoma patients. (DOCX 15 kb)
Additional file 2: Figure S1. Evaluation of the autophagy marker LC3B in esophageal cancer cell lines following 5-fluorouracil (5-FU) treatment. Untreated and treated (40 μM 5-FU for 48 h) (**A**) OE21 and (**B**) KYSE450 cells were prepared as agrose cell pellets which were fixed, processed and stained by standard immunohistochemistry. Mild staining of LC3B is detected before and after treatment in OE21 cells, while in KYSE450 cells, staining is mild in pre-treatment sections, with strong staining observed following treatment (magnification 100×). Untreated and treated (40 μM 5-FU for 48 h) (**C**) OE21 and (**D**) KYSE450 cells were fixed and stained for LC3B. Immunofluorescence analysis of OE21 cells shows little if any staining with anti-LC3B, either pre- or post-treatment. In contrast, a small number of KYSE450 cells display LC3B staining, prior to treatment, while the extent and intensity of LC3B staining is significantly increased post treatment (magnification 400×). (Cytospins were fixed in 4 % PFA for 20 min and washed with PBS. Permeabilization was carried out with 0.2 % Triton X prior to staining with anti-LC3B). (TIFF 2351 kb)

## References

[1] Jemal A, Siegel R, Ward E, Murray T, Xu J, Thun MJ (2007). Cancer statistics, 2007. CA Cancer J Clin.

[2] Siewert JR, Lordick F, Ott K, Stein HJ, Weber WA, Becker K (2007). Induction chemotherapy in Barrett cancer: influence on surgical risk and outcome. Ann Surg.

[3] Koppert LB, Wijnhoven BP, van Dekken H, Tilanus HW, Dinjens WN (2005). The molecular biology of esophageal adenocarcinoma. J Surg Oncol.

[4] Yoon HH, Forastiere AA (2008). Locally advanced esophageal adenocarcinoma: current standards and molecular predictors of outcome. Future Oncol.

[5] Yoon HH, Catalano PJ, Murphy KM, Skaar TC, Philips S, Powell M (2011). Genetic variation in DNA-repair pathways and response to radiochemotherapy in esophageal adenocarcinoma: a retrospective cohort study of the Eastern Cooperative Oncology Group. BMC Cancer.

[6] Fields RC, Strong VE, Gonen M, Goodman KA, Rizk NP, Kelsen DP (2011). Recurrence and survival after pathologic complete response to preoperative therapy followed by surgery for gastric or gastrooesophageal adenocarcinoma. Br J Cancer.

[7] Juhasz G, Neufeld TP (2006). Autophagy: a forty-year search for a missing membrane source. PLoS Biol.

[8] Mizushima N (2007). Autophagy: process and function. Genes Dev.

[9] Glick D, Barth S, Macleod KF (2010). Autophagy: cellular and molecular mechanisms. J Pathol.

[10] Jin S, White E (2008). Tumor suppression by autophagy through the management of metabolic stress. Autophagy.

[11] Lum JJ, DeBerardinis RJ, Thompson CB (2005). Autophagy in metazoans: cell survival in the land of plenty. Nat Rev Mol Cell Biol.

[12] Mathew R, Karantza-Wadsworth V, White E (2007). Role of autophagy in cancer. Nat Rev Cancer.

[13] Kondo Y, Kanzawa T, Sawaya R, Kondo S (2005). The role of autophagy in cancer development and response to therapy. Nat Rev Cancer.

[14] Lum JJ, Bauer DE, Kong M, Harris MH, Li C, Lindsten T (2005). Growth factor regulation of autophagy and cell survival in the absence of apoptosis. Cell.

[15] Vousden KH, Ryan KM (2009). p53 and metabolism. Nat Rev Cancer.

[16] Carew JS, Nawrocki ST, Kahue CN, Zhang H, Yang C, Chung L (2007). Targeting autophagy augments the anticancer activity of the histone deacetylase inhibitor SAHA to overcome Bcr-Abl-mediated drug resistance. Blood.

[17] Crowley LC, Elzinga BM, O'Sullivan GC, McKenna SL (2011). Autophagy induction by Bcr-Abl-expressing cells facilitates their recovery from a targeted or nontargeted treatment. Am J Hematol.

[18] Amaravadi RK (2008). Autophagy-induced tumor dormancy in ovarian cancer. J Clin Invest.

[19] Abedin MJ, Wang D, McDonnell MA, Lehmann U, Kelekar A (2007). Autophagy delays apoptotic death in breast cancer cells following DNA damage. Cell Death Differ.

[20] Katayama M, Kawaguchi T, Berger MS, Pieper RO (2007). DNA damaging agent-induced autophagy produces a cytoprotective adenosine triphosphate surge in malignant glioma cells. Cell Death Differ.

[21] O'Donovan TR, O'Sullivan GC, McKenna SL (2011). Induction of autophagy by drug-resistant esophageal cancer cells promotes their survival and recovery following treatment with chemotherapeutics. Autophagy.

[22] Michaud M, Martins I, Sukkurwala AQ, Adjemian S, Ma Y, Pellegatti P (2011). Autophagy-dependent anticancer immune responses induced by chemotherapeutic agents in mice. Science.

[23] Weiner LM, Lotze MT (2012). Tumor-cell death, autophagy, and immunity. N Engl J Med.

[24] Sivridis E, Giatromanolaki A, Zois C, Koukourakis MI (2010). The “stone-like” pattern of autophagy in human epithelial tumors and tumor-like lesions: an approach to the clinical outcome. Autophagy.

[25] Liao W, Sun L, Wang C, Huang H, Liu J, Liao W (2014). LC3A-positive “stone-like” structures predict an adverse prognosis of gastric cancer. Anatomical record (Hoboken, NJ : 2007).

[26] Fujii S, Mitsunaga S, Yamazaki M, Hasebe T, Ishii G, Kojima M (2008). Autophagy is activated in pancreatic cancer cells and correlates with poor patient outcome. Cancer Sci.

[27] Yoshioka A, Miyata H, Doki Y, Yamasaki M, Sohma I, Gotoh K (2008). LC3, an autophagosome marker, is highly expressed in gastrointestinal cancers. Int J Oncol.

[28] Hao CL, Li Y, Yang HX, Luo RZ, Zhang Y, Zhang MF (2014). High level of microtubule-associated protein light chain 3 predicts poor prognosis in resectable esophageal squamous cell carcinoma. Int J Clin Exp Pathol.

[29] Aoki H, Kondo Y, Aldape K, Yamamoto A, Iwado E, Yokoyama T (2008). Monitoring autophagy in glioblastoma with antibody against isoform B of human microtubule-associated protein 1 light chain 3. Autophagy.

[30] Choi JH, Cho YS, Ko YH, Hong SU, Park JH, Lee MA (2014). Absence of autophagy-related proteins expression is associated with poor prognosis in patients with colorectal adenocarcinoma. Gastroenterology research and practice.

[31] Choi J, Jung W, Koo JS (2013). Expression of autophagy-related markers beclin-1, light chain 3A, light chain 3B and p62 according to the molecular subtype of breast cancer. Histopathology.

[32] He Y, Zhao X, Subahan NR, Fan L, Gao J, Chen H (2014). The prognostic value of autophagy-related markers beclin-1 and microtubule-associated protein light chain 3B in cancers: a systematic review and meta-analysis. Tumour biology : the journal of the International Society for Oncodevelopmental Biology and Medicine.

[33] Sivridis E, Koukourakis MI, Zois CE, Ledaki I, Ferguson DJ, Harris AL (2010). LC3A-positive light microscopy detected patterns of autophagy and prognosis in operable breast carcinomas. Am J Pathol.

[34] Sivridis E, Giatromanolaki A, Liberis V, Koukourakis MI (2011). Autophagy in endometrial carcinomas and prognostic relevance of ‘stone-like’ structures (SLS): what is destined for the atypical endometrial hyperplasia?. Autophagy.

[35] Karpathiou G, Sivridis E, Koukourakis MI, Mikroulis D, Bouros D, Froudarakis ME (2011). Light-chain 3A autophagic activity and prognostic significance in non-small cell lung carcinomas. Chest.

[36] Sivridis E, Giatromanolaki A, Karpathiou G, Karpouzis A, Kouskoukis C, Koukourakis MI (2011). LC3A-positive “stone-like” structures in cutaneous squamous cell carcinomas. Am J Dermatopathol.

[37] Sivridis E, Koukourakis MI, Mendrinos SE, Touloupidis S, Giatromanolaki A (2012). Patterns of autophagy in urothelial cell carcinomas-the significance of “stone-like” structures (SLS) in transurethral resection biopsies. Urol Oncol.

[38] Giatromanolaki A, Koukourakis MI, Harris AL, Polychronidis A, Gatter KC, Sivridis E (2010). Prognostic relevance of light chain 3 (LC3A) autophagy patterns in colorectal adenocarcinomas. J Clin Pathol.

[39] Koukourakis MI, Giatromanolaki A, Zois CE, Sivridis E (2013). LC3 immunostaining pitfalls. Histopathology.

[40] Lee YJ, Ha YJ, Kang YN, Kang KJ, Hwang JS, Chung WJ (2013). The autophagy-related marker LC3 can predict prognosis in human hepatocellular carcinoma. PLoS One.

[41] Xi SY, Lu JB, Chen JW, Cao Y, Luo RZ, Wu QL (2013). The “stone-like” pattern of LC3A expression and its clinicopathologic significance in hepatocellular carcinoma. Biochem Biophys Res Commun.

[42] Bai H, Inoue J, Kawano T, Inazawa J (2012). A transcriptional variant of the LC3A gene is involved in autophagy and frequently inactivated in human cancers. Oncogene.

