# Correction to: LC3B globular structures correlate with survival in esophageal adenocarcinoma

**DOI:** 10.1186/s12885-019-6410-x

**Published:** 2019-12-03

**Authors:** Shereen El-Mashed, Tracey R. O’Donovan, Elaine W. Kay, Ayat R. Abdallah, Mary-Clare Cathcart, Jacintha O’Sullivan, Anthony O’Grady, John Reynolds, Seamus O’Reilly, Gerald C. O’Sullivan, Sharon L. McKenna

**Affiliations:** 1 Leslie C. Quick Laboratory, Cork Cancer Research Centre, BioSciences Institute, University College, Cork, Ireland; 2 Department of Pathology, Royal College of Surgeons Ireland (RCSI), Beaumont Hospital, Dublin, Ireland; 3 National Liver Institute, Menoufiya University, Shebin El Kom, Egypt; 4 Department of Surgery & Trinity Centre for Health Sciences, St James Hospital, Dublin, Ireland; 5 Department of Oncology, Cork University Hospital, Cork, Ireland

**Correction to: BMC Cancer**

**https://doi.org/10.1186/s12885-015-1574-5**

Following publication of the original article [1], the authors reported an omission in the affiliations. Dr. Shereen El-Mashed should be affiliation to both 1 and 3. This is reflected in this correction article.
