# Supplementary material for: LC3B globular structures correlate with survival in esophageal adenocarcinoma
Source: BMC Cancer. 2015 Aug 12;15:582. doi: 10.1186/s12885-015-1574-5 (PMC4533787; doi:10.1186/s12885-015-1574-5)
Supplement: Additional file 1: Table S1. — Clinical and histopathological data from both Group 1 (neoadjuvant-naïve) and Group 2 (neoadjuvant therapy) esophageal adenocarcinoma patients. (DOCX 15 kb) [file 12885_2015_1574_MOESM1_ESM.docx]

|  | **St. James's Hospital, Dublin, Ireland** | | | **Mercy University Hospital, Cork, Ireland** | |
| --- | --- | --- | --- | --- | --- |
|  | **Group 1 patients**  **(neoadjuvant-naïve)** | **Group 2 (neoadjuvant therapy)** | | **Group 1 patients**  **(neoadjuvant-naïve)** | **Group 2 (neoadjuvant therapy)** |
| **Gender** | | | | | |
| Male | 69 (82.1%) | 14 (77.8%) | 14 (70.0%) | | 24 (80.0%) |
| Female | 15 (17.9%) | 4 (22.2%) | 6 (30.0%) | | 6 (20.0%) |
| **Age** | | | | | |
| < 60 years old | 20 (23.8%) | 8 (44.4%) | 4 (20.0%) | | 11 (36.7%) |
| > 60 years old | 64 (76.2%) | 10 (55.6%) | 16 (80.0%) | | 19 (63.3%) |
| **Differentiation** | | | | | |
| Well | 9 (10.7%) | 0 (0%) | 2 (10.0%) | | 1 (3.3%) |
| Moderate | 46 (54.8%) | 9 (50.0%) | 10 (50.0%) | | 20 (66.7%) |
| Poor | 29 (34.5%) | 9 (50.0%) | 8 (40.0%) | | 9 (30.0%) |
| **Tumor staging** | | | | | |
| Stage I | 14 (16.7%) | 1 (5.6%) | 3 (15.0%) | | 1 (3.3%) |
| Stage II | 21 (25.0%) | 7 (38.9%) | 8 (40.0%) | | 11 (36.7%) |
| Stage III-IV | 49 (58.3%) | 10 (55.6%) | 9 (45.0%) | | 18 (60.0%) |
| **Lymphatic mets** | | | | | |
| Negative | 25 (29.8%) | 6 (33.3%) | 9 (45.0%) | | 7 (23.3%) |
| Positive | 59 (70.2%) | 12 (66.7%) | 11 (55.0%) | | 23 (76.7%) |
| **Vascular invasion** | | | | | |
| Negative | 46 (54.8%) | 12 (66.7%) | 17 (85.0%) | | 20 (66.7%) |
| Positive | 38 (45.2%) | 6 (33.3%) | 3 (15.0%) | | 10 (33.3%) |
| **Neural invasion** | | | | | |
| Negative | 56 (66.7%) | 10 (55.6%) | 19 (95.0%) | | 22 (73.3%) |
| Positive | 28 (33.3%) | 8 (44.4%) | 1 (5.0%) | | 8 (26.7%) |

**Supplementary Table 1**. Clinical and histopathological data from both Group 1 (neoadjuvant-naïve) and Group 2 (neoadjuvant therapy) esophageal adenocarcinoma patients.

**Supplementary Figure Legend**

**Figure S1.** Evaluation of the autophagy marker LC3B in esophageal cancer cell lines following 5-fluorouracil (5-FU) treatment. Untreated and treated (40 µM 5-FU for 48 hours) (**A**) OE21 and (**B**) KYSE450 cells were prepared as agrose cell pellets which were fixed, processed and stained by standard immunohistochemistry. Mild staining of LC3B is detected before and after treatment in OE21 cells, while in KYSE450 cells, staining is mild in pre-treatment sections, with strong staining observed following treatment (magnification 100x). Untreated and treated (40 µM 5-FU for 48 hours) (**C**) OE21 and (**D**) KYSE450 cells were fixed and stained for LC3B. Immunofluorescence analysis of OE21 cells shows little if any staining with anti-LC3B, either pre- or post-treatment. In contrast, a small number of KYSE450 cells display LC3B staining, prior to treatment, while the extent and intensity of LC3B staining is significantly increased post treatment (magnification 400x). (Cytospins were fixed in 4 % PFA for 20 minutes and washed with PBS. Permeabilization was carried out with 0.2 % Triton X prior to staining with anti-LC3B).
