# Supplementary figures and images for: LC3B globular structures correlate with survival in esophageal adenocarcinoma
Source: BMC Cancer. 2015 Aug 12;15:582. doi: 10.1186/s12885-015-1574-5 (PMC4533787; doi:10.1186/s12885-015-1574-5)

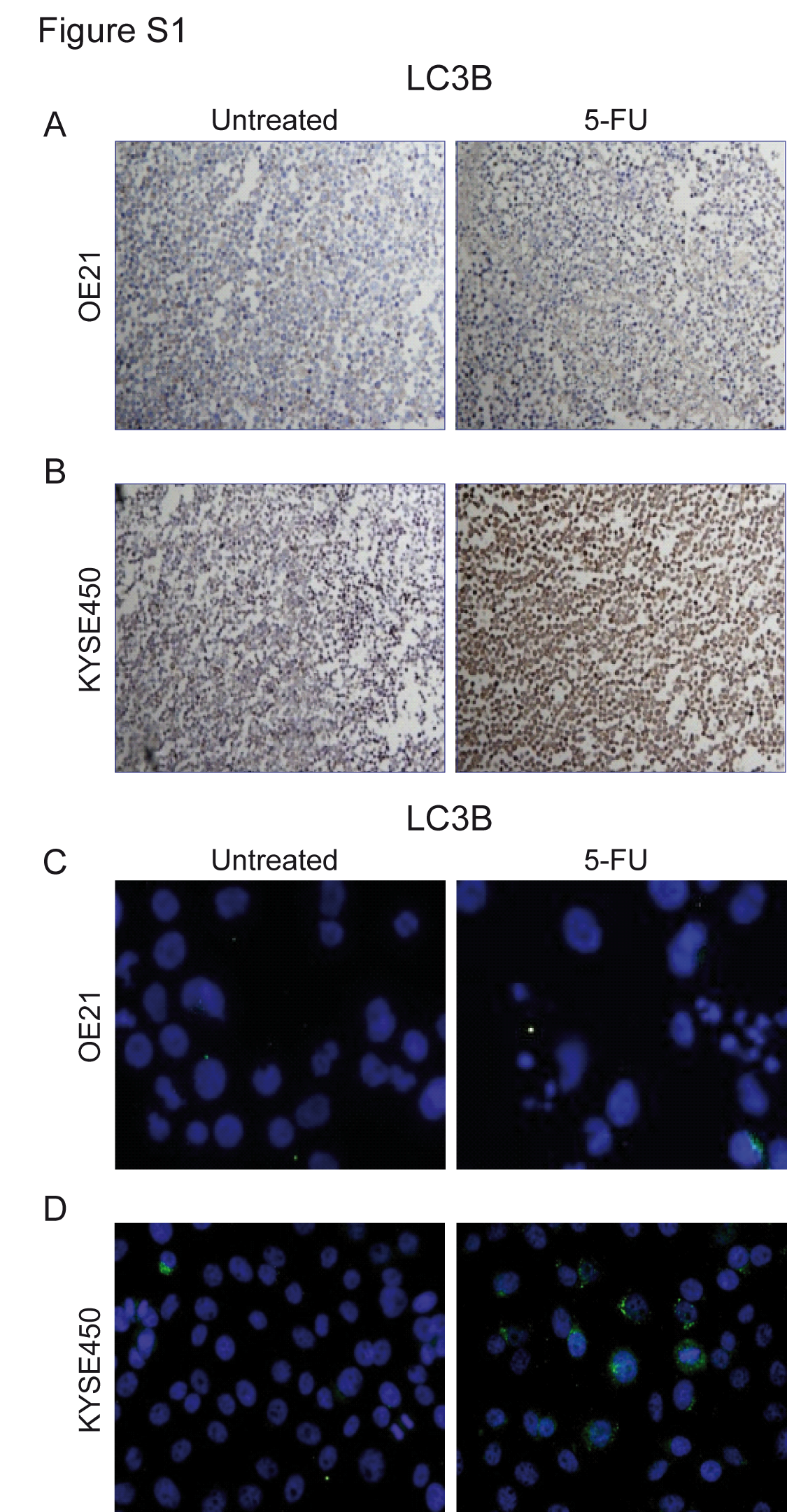

Supplement: Additional file 2: Figure S1. — Evaluation of the autophagy marker LC3B in esophageal cancer cell lines following 5-fluorouracil (5-FU) treatment. Untreated and treated (40 μM 5-FU for 48 h) (A) OE21 and (B) KYSE450 cells were prepared as agrose cell pellets which were fixed, processed and stained by standard immunohistochemistry. Mild staining of LC3B is detected before and after treatment in OE21 cells, while in KYSE450 cells, staining is mild in pre-treatment sections, with strong staining observed following treatment (magnification 100×). Untreated and treated (40 μM 5-FU for 48 h) (C) OE21 and (D) KYSE450 cells were fixed and stained for LC3B. Immunofluorescence analysis of OE21 cells shows little if any staining with anti-LC3B, either pre- or post-treatment. In contrast, a small number of KYSE450 cells display LC3B staining, prior to treatment, while the extent and intensity of LC3B staining is significantly increased post treatment (magnification 400×). (Cytospins were fixed in 4 % PFA for 20 min and washed with PBS. Permeabilization was carried out with 0.2 % Triton X prior to staining with anti-LC3B). (TIFF 2351 kb) [file 12885_2015_1574_MOESM2_ESM.tiff]
